# Cross-Generational Transmission of Early Life Stress Effects on HPA Regulators and Bdnf Are Mediated by Sex, Lineage, and Upbringing

**DOI:** 10.3389/fnbeh.2019.00101

**Published:** 2019-05-09

**Authors:** Elena J. L. Coley, Camila Demaestri, Prabarna Ganguly, Jennifer A. Honeycutt, Shayna Peterzell, Natasha Rose, Nida Ahmed, Mary Holschbach, Malav Trivedi, Heather C. Brenhouse

**Affiliations:** ^1^Developmental Neuropsychobiology Laboratory, Department of Psychology, Northeastern University, Boston, MA, United States; ^2^Neural Metabolism and Epigenetics Laboratory, Department of Pharmaceutical Sciences, Nova Southeastern University, Fort Lauderdale, FL, United States; ^3^Department of Behavioral Neuroscience, College of Psychology, Nova Southeastern University, Fort Lauderdale, FL, United States

**Keywords:** methylation, maternal separation (MS), corticosterone, rat, sex, anxiety

## Abstract

Early life stress (ELS) is a potent developmental disruptor and increases the risk for psychopathology. Various forms of ELS have been studied in both humans and rodents, and have been implicated in altered DNA methylation, gene transcription, stress hormone levels, and behavior. Although recent studies have focused on stress-induced epigenetic changes, the extent to which ELS alters HPA axis function and stress responsivity across generations, whether these effects are sex-specific, and how lineage interacts with upbringing to impact these effects, remain unclear. To address these points, two generations of rodents were utilized, with the first generation subjected to ELS via maternal separation, and the second to a balanced cross-fostering paradigm. We hypothesized that ELS would disrupt normative development in both generations, manifesting as altered methylation and expression of genes associated with stress signaling pathways (Nr3c1, Nr3c2, and Bdnf), blunted corticosterone (CORT), and anxiety-like behaviors. Additionally, we expected deficits in the second generation to be modulated by caretaking environment and for the pattern of results to differ between the sexes. Results suggest that direct exposure to ELS leads to sex-specific effects on gene regulation and HPA functioning in adulthood, with maternal separation leading to increases in Bdnf methylation in both sexes, decreases in Bdnf expression in females, and decreases in Nr3c1 methylation in males, as well as blunted CORT and less anxiety-like behavior in females. These alterations converged with caretaking to impart perturbations upon the subsequent generation. Across sex, ELS lineage led to decreased methylation of Nr3c1, and increased methylation of Bdnf. In fostered animals, upbringing by a previously stressed mother interacted with offspring lineage to impact methylation of Nr3c1 and Bdnf. Upbringing was also implicated in altered anxiety-like behavior in males, and baseline CORT levels in females. Such effects may correspond with observed alterations in maternal behavior across groups. In conclusion, ELS conferred enduring sex-specific alterations, both first-hand and *trans*-generationally via lineage and upbringing. Importantly, lineage of cross-fostered pups was sufficient to normalize or disturb maternal behavior of foster-dams, an observation requiring further elucidation. These results have implications for multi-generational effects of ELS in humans and may motivate early interventions.

## Introduction

Every generation of each geographic, cultural, and political group across the world is met with conflicts, struggles, and environmental stressors. Stress is an expected part of everyday life and in acute doses can be adaptive (McEwen, 2008) or foster resilience (Majcher-Maslanka et al., 2018). However, chronic stress, particularly during critical periods of development, can pathologically disrupt normative maturation (O’Mahony et al., 2009; Franklin et al., 2010; van der Knaap et al., 2014; Ganguly and Brenhouse, 2015; Romens et al., 2015). Stress that occurs early in life (early life stress; ELS) can have long-lasting effects on physiology and behavior (Shonkoff et al., 2012; Brunton, 2015). For example, repeated postnatal maternal separation, a well-characterized model of ELS, disrupts development and gives rise to morphological, neurochemical, and behavioral deficits in rodents that appear to be analogous to those in humans exposed to childhood adversity (Lehmann and Feldon, 2000; Franklin et al., 2010; Ganguly and Brenhouse, 2015). Importantly, developmental effects of adversity in children of one generation can manifest in their descendants, via altered parental behaviors and/or epigenetics (Cowan et al., 2016; Scorza et al., 2018). The current study assessed the impact of ELS via maternal separation on rodents and their offspring to investigate potential impacts of ELS lineage and parenting from one generation to another.

Phenotypic consequences of ELS can originate from molecular changes such as DNA methylation (Romens et al., 2015; Turecki and Meaney, 2016) and altered gene transcription (Vazquez et al., 1996), which have effects at the endocrine level, including dysregulation of stress hormones (O’Mahony et al., 2009; Magalhaes et al., 2018). The hypothalamic-pituitary-adrenal (HPA) axis is particularly susceptible to stress-driven alterations (Vazquez et al., 1996; van der Knaap et al., 2014; Romens et al., 2015). HPA axis regulation is mediated largely by glucocorticoids such as corticosterone (CORT) in rats, and corresponding glucocorticoid (GR) and mineralocorticoid (MR) receptors. GRs and MRs reside throughout the central and peripheral nervous systems (Joels and Baram, 2009), with particularly high expression in the hippocampus, which is implicated in both appraisal processes and stress adaptation (de Kloet et al., 2005). In humans exposed to childhood adversity, epigenetic modifications of the Nr3c1 GR-encoding genes are strongly correlated with increased vulnerability to attachment disorders such as borderline-personality disorder (Radtke et al., 2015). In rats, the use of several types of ELS procedures has yielded similar results. For example, one study using maternal separation reported reduced hippocampal GRs via heightened methylation at the promoter region (Zhu et al., 2017). MRs, encoded for by the gene Nr3c2, are another possible target for ELS-induced dysfunction of the HPA axis (de Kloet et al., 2005), and in fact are reportedly reduced following ELS (Vazquez et al., 1996).

Developmental vulnerability following ELS is likely associated with the fact that HPA dysregulation can result in reduced synaptic connectivity and dendritic atrophy (Daskalakis et al., 2015). Growing evidence suggests that stress-induced loss of trophic support is due to actions of stress hormones on brain-derived neurotrophic factor (BDNF). Indeed, ELS increases methylation and reduces expression of Bdnf in rats, which may have critical consequences for pathological modifications in plasticity (Roth and Sweatt, 2011). Glucocorticoid signaling pathways often co-exist with those of neurotrophin signaling, and together GR and BDNF systems act in antagonistic as well as synergistic manners to regulate stress responsivity. Here, we investigated effects of ELS on methylation and expression of Nr3c1, Nr3c2, and Bdnf.

This ELS-induced dysregulation of key molecular and hormonal players in the HPA axis (Vazquez et al., 1996; van der Knaap et al., 2014; Romens et al., 2015) and subsequently, neurotrophic signaling (Roth and Sweatt, 2011) may increase susceptibility to a variety of behavioral dysfunctions later in life (O’Mahony et al., 2009; Desgent et al., 2012; Juruena et al., 2015). Studies in rodents have largely reported increased anxiety (Liu et al., 2017; Bondar et al., 2018) and cognitive deficits (Bath et al., 2017; Liu et al., 2017), with robust sex differences. Importantly, ELS also has been found to yield spontaneous behavioral resilience, of unknown etiology (Liu et al., 2017). Here, we explored the extent to which observed genetic and endocrine effects manifest in behavior by utilizing an open field test (OFT) paradigm, and measuring the CORT response following the mildly stressful exposure to an open field arena (Spencer and Deak, 2017).

Notably, while converging evidence is expanding our understanding about the effects of ELS during one lifetime, there is a paucity of knowledge about how epigenetics or upbringing can lead to altered baseline HPA function in males and females of subsequent generations. We therefore examined two generations in this study. Furthermore, it is unclear the degree to which cross-generational transmission is due to (epi)genetic inheritance or altered early life/parenting environment, both of which have been demonstrated to alter later exploratory behaviors, stress reactivity, and sensorimotor functioning (Pedersen et al., 2011). Clarity on this issue may be provided by utilizing a cross-fostering paradigm, a method that has been implemented to examine the effects of maternal behavior on offspring (Pometlova et al., 2009; Pedersen et al., 2011). Cross-fostering was applied in this study to differentiate effects of inherited epigenetic pathologies from those of upbringing.

The aim of the current study is to elucidate sex-specific epigenetic, behavioral, and endocrine effects of ELS, and the extent to which these effects are transmitted across generations. We hypothesized that, if ELS disrupts stress responsivity *trans*-generationally via altered hippocampal Nr3c1, Nr3c2, or Bdnf expression, then adult rats with a history of ELS, as well as their offspring, will display atypical methylation and expression in our genes of interest, blunted corticosterone following a mild acute stressor, and increased anxiety-like behaviors. Furthermore, we investigated whether these effects are influenced by upbringing. We hypothesized that being raised by an ELS dam will compound, and being raised by a control dam will ameliorate, any stress-induced genetic or epigenetic inherited alterations. Finally, we explored sex differences in response to early life stress (both directly, and second-hand). We hypothesized that females will be more susceptible to stress dysregulation than males. Findings from this study will increase our understanding of the ways that early life adversity contribute to pathology later in life. Clarifying these mechanisms may inform efforts toward intervention in, and prevention of, relevant physiological and psychological conditions.

## Materials and Methods

### Animals

Throughout all procedures, animals were housed in a temperature- and humidity- controlled vivarium (22 ± 2°C), with a 12-h light/dark cycle (lights period 0700–1900 h) and *ad libitum* access to food and water. Unless otherwise specified, all animals were left undisturbed in their cages except for normal husbandry procedures (including weekly cage cleanings and weights at Postnatal Day [PD] 9 and 20). All experiments were performed in accordance with the 1996 Guide for the Care and Use of Animals and were approved by the Northeastern University Animal Care and Use Committee.

### First Filial (F1) Generation – Maternal Separations

Primiparous adult (PD 70–90) Sprague-Dawley rats purchased from Charles River Laboratories (Wilmington, MA, United States) were mated in-house for F1 of this study. Breeding was accomplished via harem pairing (one male housed with two females) until confirmation of pregnancy. Male breeders were not re-used, in order to avoid extraneous effects of sexual experience (Beloate and Coolen, 2018). To confirm successful mating, vaginal swabs were taken from each female every morning to check for presence of sperm. If sperm was observed, pregnancy was assumed and pregnant dams were single-housed. All dams were single-housed until they gave birth, with date of birth marked PD0. Each litter was then randomly assigned to one of two treatment conditions: ELS or control (Con). On PD1, litters were culled to 10 pups per cage (±2), with equal males and females whenever possible. From PD2–PD10, ELS pups were isolated in a separate room from the dam 4 h/day beginning at 0900 in individual cups with home-nest shavings. The cups were secured in a shallow, circulating water bath set to 35°C. From PD11–PD20, ELS pups were isolated in standard mouse cages with home bedding, since these days coincide with rat pups beginning to self-regulate their body temperature. During this separation paradigm, pups were deprived of maternal tactile stimulation and nursing. However, the pups could still sense maternal odor, and maintained nest temperature. Correspondingly, dams of ELS pups remained in their home cages and were deprived of their litters during the separation period. Con pups were left undisturbed except for normal husbandry procedures and weighing at PD 9 and PD20. At PD21, all pups were weaned and pair-housed with same-sex and -condition littermates. Animals were left undisturbed apart from husbandry from PD21 to PD60.

### F2 Generation – Breeding and Fostering

At PD60, two males and two females were taken from each of six Con and six ELS F1 litters for breeding. To test for effects of lineage on a second generation, one male and one female from two different litters of the same treatment condition were bred. Pairs were housed together until confirmation of pregnancy via sperm checks. After successful mating, all males were removed from the cage and dams were single-housed until they gave birth. Three females failed to become pregnant, and three litters were excluded due to unusually small number of pups or highly skewed male to female ratio. To test for effects of upbringing, at PD0, each of the 18 F2 litters was assigned to an environmental condition (3 L per condition): pups raised by their own biological mother (Con Bio or ELS Bio), pups cross-fostered to another dam reared in the same condition as the biological mother (Con → Con or ELS → ELS), or pups cross-fostered to another dam reared in a different condition from the biological mother (ELS → Con or Con → ELS). See Table 1 for clarification of F2 designations. At PD1, litters were culled to 10 pups per cage (±2), with equal males and females whenever possible. Within fostering conditions, whole litters were removed from their biological dam and placed into a cage with the foster dam. All F2 litters were then left undisturbed except for normal husbandry procedures. At PD21, pups were weaned from the dams, and pair-housed within sex and condition in new cages. From PD21 to PD60, animals of all condition were left undisturbed.

**Table 1:** F2 group designations.

Designation	Biological mother	Fostered to
Bio Con	Con	Not fostered
Bio ELS	ELS	Not fostered
Con → Con	Con	Con
ELS → ELS	ELS	ELS
Con → ELS	Con	ELS
ELS → Con	ELS	Con

### Maternal Behavior

At two ages (PD6–7 and PD13–14), maternal behavior toward F2 litters was recorded using CCTV for 30 min at four time points (1430, 2330, 0400, and 0830 h) and analyzed for a representative sample of maternal behavior. Recordings done during the dark cycle were illuminated with dim red light (8 lux). Videos were manually analyzed by a naive experimenter for both duration and frequency of the following behaviors: Spending time on/off nest, nursing (active [high crouch] and passive [low crouch and supine]), and licking/grooming pups. Total duration, frequency of bouts, and duration/bout over all four recordings were recorded.

### Open Field Test (OFT) Behavior

Between PD60 and PD69, animals from each generation (*n* = 6–8; 1–2 pups/sex/litter) were tested for open field activity in a 100 cm × 100 cm arena with opaque black floor and sides, in a room with equal illumination to the animal facility where animals were housed (∼25 lux). The arena was cleaned with 40% ethanol between each animal. Behavior was recorded using CCTV. Animals were placed into a corner of the arena and allowed to explore freely for 5 min, before being removed from the arena. Analysis was conducted using EthoVision 9.0 software (Noldus). Measures of interest included time spent in each of three zones (perimeter, inside, and center), frequency of visits to the center, latency to first entry into center, and overall distance and velocity. Due to differences between groups for total distance traveled, all subsequent measures were corrected by dividing by total distance traveled. For this reason, units are all in the format x/cm.

After the conclusion of the OFT, but before blood collection (below), the animal was removed from the arena, and in order to obtain data for a separate study, a con-specific (sex- and age-matched) animal was placed under a small metal cage in a corner of the arena as part of a separate experiment. The test animal was replaced in the arena, and was allowed to interact with the conspecific (separated by the small cage) for five additional minutes.

### Blood Corticosterone

#### Baseline Collection

Twenty-four hours before behavioral testing, blood samples were collected from 6 to 8 animals/sex/group from the saphenous vein between 0900–1130 h for baseline CORT analysis. Rats were briefly (2–3 min) anesthetized with isoflurane to allow for the animal to be comfortably restrained by hand. Fur from the lower portion of the hind leg(s) was shaved, and petroleum-based ointment was gently rubbed on the collection area for increased visualization of the saphenous vein. A sterile lancet was used to prick the saphenous vein and approximately 500 l of blood were collected per animal in a non-coated 1.5 mL microfuge tube. Blood was left to clot for 1 h at room temperature and was then centrifuged [1,000 ×*g* (RCF)] at 4°C for 10 min. Serum (50–150 l) was collected and immediately stored at -20°C for later analysis.

#### Test Collection

Thirty minutes after the conclusion of the conspecific exposure, animals (*n* = 8/sex/group) were briefly anesthetized with isoflurane and then sacrificed by rapid-decapitation. Approximately 1 mL trunk blood was collected in microfuge tubes, and was treated identically to saphenous blood samples.

#### Corticosterone Analysis

Total CORT levels were determined via a highly sensitive corticosterone ELISA kit (Enzo Life Sciences; CAT. No.: ADI-901-097), run per the manufacturer’s instructions. Serum samples were combined with 1:100 steroid displacement solution, vortexed and allowed to sit for 5 min before the addition of included buffer solution to make a 1:40 serum solution, which was used according to instructions. All samples were run in triplicate, including blanks and controls. Following completion of incubation and washing steps, ELISA plates were read to determine optical density on an Opsys MR plate reader (Dynex Technologies) at 405 nm. The mean optical density for each sample was calculated based on a standard curve, generated from standardized CORT dilutions, with analyses conducted via freely available online software^1^ to obtain the concentration of CORT (in pg/mL) within each sample.

### Brain Tissue Analyses

After sacrifice and trunk blood collection, brains were extracted and right and left dorsal hippocampus tissue was hand-dissected using a brain atlas (Paxinos and Watson, 1998) as a guide, collected in microfuge tubes and stored at -80°C until RNA isolation procedures.

#### Bisulfite Sequencing

Bisulfite sequencing was run on dorsal hippocampus tissue (*n* = 3–4, subjects from different litters were chosen randomly from each group). DNA from the same tissue was used to quantify methylation in the promoter regions of two genes: Nr3c1 (exon 17, CpG sites 1, 2, 3, 4, and 5) and Bdnf (CpG sites -35, -23, 19, 35, and 43). Since gene expression measurement of Nr3c2 was added after bisulfite sequencing was completed, bisulfite sequencing was not performed for the Nr3c2 gene. The bisulfite conversion and pyrosequencing were conducted as described previously (Trivedi et al., 2014). Briefly, for bisulfite conversion, the extracted DNA (500 ng; concentration: 50 ng/ml) was treated with the EZ DNA Methylation-Gold Kit (Zymo Research, Orange, CA, United States) according to the manufacturer’s protocol. 30 μL of M-Elution Buffer (Zymo Research) was used for the final elution. Methylation of DNA was quantified with bisulfite treatment of DNA and simultaneous polymerase chain reaction (PCR), using primers and conditions previously described for Bdnf exon IV promoter region (Boersma et al., 2014) or commercially purchased for Nr3c1 from Qiagen (Rn_Nr3c1_01_PM PyroMark CpG assay; PM00508074). Results were reported as the percent of the sum total of methylated and unmethylated cytosines that consisted of 5-methylated cytosines (%5mC). Additionally, non-CpG cytosine residues were used as internal controls to validate bisulfite conversion. The Pyrosequencing-based assay can evaluate individual measures of methylation at more than one CpG dinucleotide. All samples were subjected to a quality control check incorporated in the software, which evaluates the bisulfite conversion rate of any cytosine not followed by a guanine. Nr3c1 and Bdnf methylation levels were analyzed each at five different CpG sites. Differentially methylated standard oligos were used as internal standard for sequencing efficiency. Technical replicates were also conducted to ensure the replicability of the experiments.

#### Isolation, Amplification and Relative Quantification of Gene Expression

RNA was isolated from whole dorsal hippocampus tissue (*n* = 6–8) using the RNAqueous-4PCR Total RNA Isolation Kit (Applied Biosystems, Foster City, CA, United States) and processed as indicated by the manufacturer’s instructions. cDNA was synthesized using the High Capacity cDNA Reverse Transcriptase Kits (Applied Biosystems, Foster City, CA, United States). Amplification of cDNA was performed using TaqMan Gene Expression Assays (Applied Biosystems). cDNA concentration was quantified by Nanodrop 2000 Spectrophotometer (Thermo Fisher Scientific), and samples were diluted accordingly. Gene-specific primers were purchased from Thermo Fisher Scientific for the following genes of interest: Nr3c1 (Rn.90070), Nr3c2 (Rn.9678), and Bdnf (Rn.11266). Amplification of all gene transcripts was performed in triplicate on a StepOnePlus Real-Time PCR System (Applied Biosystems) using standard cycling conditions, as recommended by the manufacturer. For each reaction, 4 μl of diluted (1:4) cDNA was placed in a 20 μl reaction plate containing 16 μl of master mix and 1× dilution of each primer (Applied Biosystems, Life Technologies, United States). Reactions were performed with an initial holding stage of 50°C for 2 min and 95°C for 10 min, followed by 35 subsequent cycles of 15 s at 95°C and 1 min at 60°C. The relative standard curve analysis method was used, with the threshold cycle (CT) (number of cycles required to reach detection threshold) determined for each reaction and the 2^-ΔΔC(t)^ method used to determine gene expression relative to the house-keeping control gene, β-actin (Thermo Fisher Scientific, AILJKN2). β-actin was selected as an endogenous control because it has been shown to be a stable reference gene within the rat cortex and hippocampus (Tanic et al., 2007). However, since only one housekeeping gene was used, coefficient of variability tests were performed to test if the housekeeping gene was stable across all experimental manipulations. Inter-plate variability was measured using three qPCR assays performed on three separate days. Intra-assay variability was measured by performing a single qPCR assay that included 10 replicates of cDNA copy number 1 × 105, 1 × 103, 1 × 102, and 1 × 101. The mean coefficient of variability was 0.7% for inter-plate assay and 0.5% for intra-plate assay, which demonstrated the repeatability of the reactions.

### Estrous Phase Monitoring

Vaginal swabs (or sham handling in males) were taken on both the day prior to and the day of behavioral testing in all subjects. Females were vaginally swabbed, and males were “sham-handled” by the experimenter briefly picking up the animal and applying gentle pressure to the genital area with a cotton-tipped applicator. Swabs were analyzed for estrous cycle, and separated into four stages: proestrus, estrus, metestrus, and diestrus based on observed cell type and count. Groups were analyzed for the breakdown of identified estrous stages to identify whether group difference could be driven by hormonal status.

### Data Analysis

Before statistical analyses, all data were run through Grubbs Outlier Test, alpha = 0.05, and significant outliers were removed. F1 methylation, gene expression, and behavior data were analyzed in PRISM 7 (Graphpad Software) with two-way ANOVAs (Sex × Treatment), alpha = 0.05. F1 corticosterone data were analyzed in SPSS Statistics 25 (IBM) with a mixed three-way [Sex × Treatment × (time)], with baseline and test as repeated measures, and then followed up in PRISM with two-way ANOVAs (Sex × Treatment) for baseline and post-test timepoints. Significant effects were followed up where indicated with *post hoc* Bonferroni’s multiple comparisons tests to determine group differences. Maternal behaviors were analyzed with one-way ANOVA to compare dam/litter pairs in different upbringing groups (Con Bio, Con → Con, ELS → Con, ELS Bio, ELS → ELS, or Con → ELS), followed by Tukey’s multiple comparison tests. Tukey’s was chosen for maternal behavior due to the large number of means being compared in *post hoc* tests. *Post hoc* tests were aimed to reveal effects of the dam’s experience alone (Con Bio vs. ELS Bio), and effects of different fostering environments for Con dams and for ELS dams. F2 growth, methylation, expression, and behavior data were analyzed in SPSS with three-way ANOVAs (Sex × Lineage × Upbringing), alpha = 0.05, with ‘Lineage’ referring to the treatment of the birth mother, and ‘Upbringing’ referring to whether the subject had been raised by its own mother, fostered within condition, or fostered across condition. F2 CORT data were analyzed in SPSS with a three-way (Sex × Lineage × Upbringing) repeated measures (baseline and test), and then followed up in PRISM with three-way ANOVAs (Sex × Lineage × Upbringing). Where indicated, the three-way ANOVAs were then followed in PRISM by two-way ANOVAs (Sex × Lineage, Lineage × Upbringing, Sex × Upbringing), and then with subsequent *post hoc* Bonferroni’s multiple comparisons tests to determine differences between groups.

### Ethics

All methods were run in conjunction with Northeastern University Department of Animal Medicine Institutional Animal Care and Use Committee, protocol #16-0516R.

## Results

### F1—Direct Effects of ELS on Offspring

#### Effects of ELS on Growth

Effects of ELS on weight gain are described in Supplementary Figure S1.

#### Effects of ELS on Gene Methylation

**FIGURE 1:**
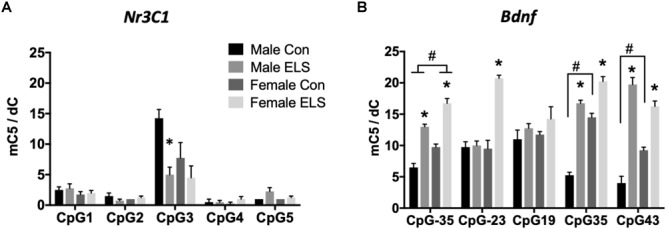
Methylation of hippocampal Nr3c1 **(A)** and Bdnf **(B)** in adulthood following maternal separation ELS or control rearing in males and females. Means ± SEM are shown. ^∗^*p* < 0.05 difference between Con and ELS within sex. ^#^*p* < 0.05 main effect of sex or *p* < 0.05 difference between male and female of same treatment group. *n* = 3–4.

Nr3c1 methylation: Two-way ANOVAs (Sex × Treatment) were run at each of five CpG sites. Significant effects were only observed at CpG site 3. A main effect of treatment (*F*_1,12_ = 11.54; *p* = 0.0053) followed by *post hoc* comparisons revealed that males exposed to ELS displayed significantly less methylation at CpG site 3 compared to Con males (*p* = 0.0079; Figure 1A).

##### Bdnf methylation

Significant effects of ELS were observed at four of five CpG sites (Figure 1B). CpG designations for the Bdnf exon IV promotor site indicate distance from transcription start site. At CpG site -35, main effects of sex (*F*_1,12_ = 35,64; *p* < 0.0001) and treatment (*F*_1,12_ = 132.5; *p* < 0.0001) and *post hoc* comparisons revealed that females displayed overall higher methylation, and ELS exposure resulted in higher methylation in both males (*p* < 0.0001) and females (*p* < 0.0001). At CpG site -23, a sex × treatment interaction (*F*_1,12_ = 37.71; *p* < 0.0001) revealed that ELS led to significantly greater methylation in females (*p* < 0.0001), but not in males. At CpG site 19, there were no significant differences for any group, *p* > 0.05. At CpG site 35, a sex × treatment interaction (*F*_1,12_ = 23; *p* = 0.0004) revealed that while ELS led to higher methylation in both males (*p* < 0001) and females (*p* < 0.0001), a higher baseline level in Con females compared to Con males (*p* < 0.0001) led to a larger effect of ELS in males. At CpG site 43, a Sex × Treatment interaction (*F*_1,12_ = 22.8; *p* = 0.0005) revealed, similarly to CpG site 35, while ELS led to higher methylation in both males (*p* < 0001) and females (*p* = 0.001), a higher baseline level in Con females compared to Con males (*p* = 0.0096) led to a larger effect of ELS in males.

#### Effects of ELS on Gene Expression

**FIGURE 2:**
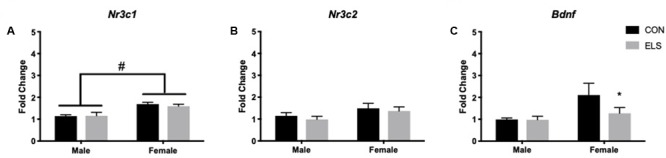
Hippocampal mRNA expression of Nr3c1 **(A)**, Nr3c2 **(B)**, and Bdnf **(C)** in adulthood following maternal separation ELS or control rearing in males and females. Means ± SEM are shown. ^∗^*p* < 0.05 difference between Con and ELS within sex. ^#^*p* < 0.05 main effect of sex or *p* < 0.05 difference between male and female of same treatment group. *n* = 6–8.

Quantitative PCR was performed on Nr3c1, Nr3c2, and Bdnf. A main effect of sex revealed that females expressed higher levels of Nr3c1 than males, regardless of treatment (*F*_1,27_ = 10.4; *p* = 0.003; Figure 2A). Sex was also found to have a trend-level main effect on Nr3c2 expression (*F*_1,27_ = 4.003; *p* = 0.055), with females again displaying higher levels than males, regardless of treatment (Figure 2B). For Bdnf, a sex × treatment interaction (*F*_1,28_ = 4.59; *p* = 0.04) revealed that ELS exposure led to lower Bdnf expression in females (*p* = 0.009), but not males (Figure 2C).

#### Effects of ELS on Open Field Behavior

**FIGURE 3:**
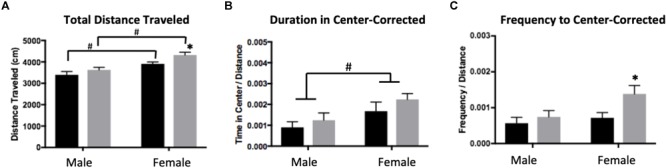
Distance traveled **(A)**, duration of time spent in the center **(B)**, and frequency of visits to the center **(C)** of an open field in adulthood following maternal separation ELS or control rearing in males and females. Means ± SEM are shown. ^∗^*p* < 0.05 difference between Con and ELS. ^#^*p* < 0.05 main effect of sex or *p* < 0.05 difference between male and female of same treatment group. *n* = 6–8.

A two-way ANOVA showed significant main effects of sex (*F*_1,28_ = 235.8, *p* < 0.0001) and treatment (*F*_1,28_ =15,26, *p* = 0.0005) for total distance traveled, with females traveling more than males and ELS subjects traveling more than Con (Figure 3A). *Post hoc* analyses revealed that within condition, females traveled farther than males (Con: *p* = 0.0092; ELS: *p* = 0.0001). Furthermore, ELS females traveled farther than Con females (*p* = 0.0072). After correcting for total distance traveled (see section “Materials and Methods”), a main effect of sex suggested that females spent more time in the center of the open field (*F*_1,28_ = 4.74, *p* = 0.038; Figure 3B), with no treatment effects. However, ELS-exposed subjects made more visits to the center than Con subjects (main effect of treatment: *F*_1,28_ = 4.213; *p* = 0.0496), which was driven by treatment effects in females (*P* = 0.037; Figure 3C). For data on uncorrected duration and frequency of center visits, see Supplementary Figure S2.

#### Effects of ELS on Corticosterone Secretion

**FIGURE 4:**
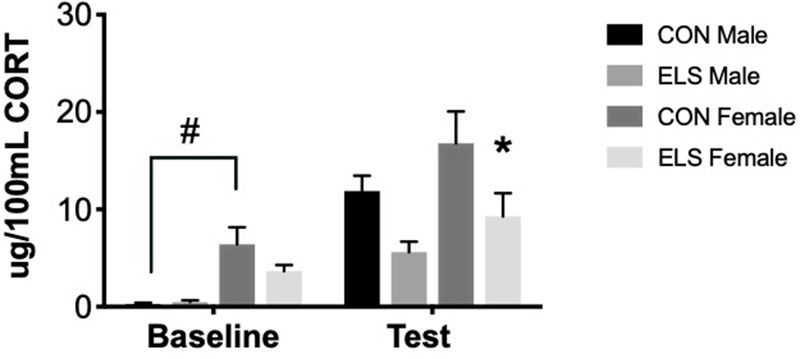
Serum corticosterone at baseline and post-exposure to an open field arena and a conspecific in male and female adults following maternal separation ELS or control rearing. Means ± SEM are shown. ^∗^*p* < 0.05 difference between Con and ELS within sex. ^#^*p* < 0.05 main effect of sex or *p* < 0.05 difference between male and female of same treatment group. *n* = 6–8.

Repeated-measures ANOVA revealed significant main effects of time (*F*_1,26_ = 13.997, *p* = 0.001), sex (*F*_1,26_ = 6.347, *p* = 0.019) and treatment (*F*_1,26_ = 5.893, *p* = 0.023). *Post hoc* analyses revealed that at baseline, Con females had significantly higher levels of CORT when compared to Con males (*p* = 0.001). After exposure to the open field arena, ELS females showed lower CORT levels when compared to Con females (*p* = 0.049), an effect not observed in males (Figure 4).

### F2—Cross-Generational Effects and Fostering

#### F2 Maternal Behavior and Growth

##### Maternal behavior

**FIGURE 5:**
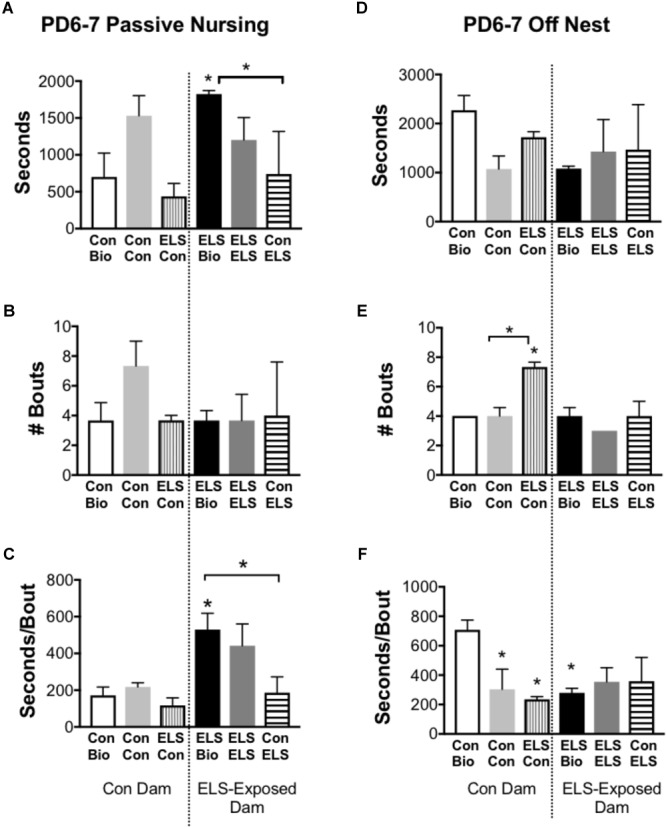
Selected maternal behaviors of mothers exposed to maternal separation ELS or control rearing as neonates. Left: Duration **(A)**, Frequency **(B)**, and Duration/Bout **(C)** of Passive Nursing Behavior. Right: Duration **(D)**, Frequency **(E)**, and Duration/Bout **(F)** of Time Spent Off Nest. Bars represent dam-litter combinations depending on fostering. Means ± SEM are shown. ^∗^*p* < 0.05 difference from Con Bio. ^∗^ with bracket *p* < 0.05 difference between designated groups. *n* = 3.

Time spent on and off the nest, active (high crouch) and passive (low crouch and supine) nursing, and licking/grooming were compared between groups at P6–7 and P13–14 (*n* = 3 dams/group). Of note, observation of maternal behaviors at four time-points per day, on two different days offered only limited information about the stability of that behavior in individual dams. No differences between groups were observed at P13–14 Supplementary Table S1B). At P6–7, measures of active nursing and licking/grooming were unaffected by group Supplementary Table S1A). The time spent in passive nursing at P6–7, however, was different between groups (*F*_5,12_ = 4.2; *p* = 0.0194; Figure 5A). ELS-exposed dams raising their biological offspring (ELS Bio) spent significantly more time in passive nursing compared to their Con counterparts (Con Bio) (*p* = 0.026) and compared to ELS dams raising pups with Con-lineage (Con → ELS; *p* = 0.026). This was driven by longer passive nursing bouts (*F*_5,12_ = 5.835; *p* = 0.0058; Figure 5C), rather than the frequency of passive nursing bouts (*p* = 0.424; Figure 5B). Specifically, ELS Bio dams engaged in longer passive nursing bouts compared to Con Bio (*p* = 0.03), and also compared to ELS dams raising fostered litters from Con mothers (Con → ELS; *p* = 0.038). However, ELS-exposed dams raising fostered litters from another ELS dam (ELS → ELS) engaged in similarly long passive nursing bouts, which did not differ from ELS Bio (*p* = 0.939). Therefore, raising pups born to a Con dam normalized atypical maternal behavior in ELS dams. While duration of time spent off the nest did not differ between groups (*p* = 0.301; Figure 5D), the number of times dams left the nest was different between groups (*F*_5,10_ = 9.4; *p* = 0.0015; Figure 5E). ELS experience of the mother did not affect this behavior, however, fostering pups with ELS lineage to Con dams significantly altered frequency of exits from the nest. In other words, Con dams left the nest more often when raising pups born to an ELS-exposed mother (ELS → Con) compared to those raising their own biological offspring (*p* = 0.011) and compared to those raising pups born to another Con mother (*p* = 0.0052). However, while ELS → Con dams left the nest more often, they spent significantly less time away during each excursion (seconds/bout) from the nest compared to Bio Con dams (*p* = 0.017). This was also true of Con → Con dams (*p* = 0.0467) and Bio ELS dams (*p* = 0.0329) (Figure 5F).

##### Growth

Effects of ELS on weight gain are described in Supplementary Figure S1.

#### F2 Gene Methylation

Due to the large number of comparisons, detailed ANOVA results for F2 gene methylation are shown in Tables 2, 3.

**Table 2:** ANOVA results for F2 Nr3c1 methylation.

		NrC31				
	Comparison	CpG1	CpG2	CpG3	CpG4	CpG5
Inter-actions	Lineage × Upbringing × Sex	*F*_2,24_ = 1.19; *p* = 0.32	*F*_2,24_ = 5.58; *p* = 0.010	*F*_2,24_ = 1.192; *p* = 0.32	*F*_2,24_ = 0.847; *p* = 0.44	*F*_2,24_ = 1.192; *p* = 0.32
	Lineage × Upbringing	*F*_2,24_ = 4.78; *p* = 0.018	*F*_2,24_ = 0.89; *p* = 0.423	*F*_2,24_ = 3.76; *p* = 0.038	*F*_2,24_ = 1.26; *p* = 0.30	*F*_2,24_ = 0.70; *p* = 0.505
	Sex × Lineage	*F*_1,24_ = 4.15; *p* = 0.0527	*F*_1,24_ = 2.2; *p* = 0.151	*F*_1,24_ = 0.04; *p* = 0.85	*F*_1,24_ = 0.89; *p* = 0.355	*F*_1,24_ = 35.1^∗∗∗^
	Sex × Upbringing	*F*_2,24_ = 3.63; *p* = 0.042	*F*_2,24_ = 0.71; *p* = 0.502	*F*_2,24_ = 3.46; *p* = 0.048	*F*_2,24_ = 0.375; *p* = 0.691	*F*_2,24_ = 0.22; *p* = 0.807
Main effects	Lineage	*F*_1,24_ = 32.0^∗∗∗^	*F*_1,24_ = 63.3^∗∗∗^	*F*_1,24_ = 45^∗∗∗^	*F*_1,24_ = 0.89; *p* = 0.355	*F*_1,24_ = 24.99^∗∗∗^
	Upbringing	*F*_2,24_ = 5.11; *p* = 0.014	*F*_2,24_ = 5.58; *p* = 0.010	*F*_2,24_ = 5.66; *p* = 0.010	*F*_2,24_ = 1.01; *p* = 0.378	*F*_2,24_ = 0.86; *p* = 0.434
	Sex	*F*_1,24_ = 0.82; *p* = 0.374	*F*_1,24_ = 1.47; *p* = 0.237	*F*_1,24_ = 13.3; *p* = 0.0013	*F*_1,24_ = 1.192; *p* = 0.32	*F*_1,24_ = 37.96^∗∗∗^
Effects of lineage	Bio Con vs. Bio ELS Male	*p* = 0.002	*p* = 0.996	*p* = 0.018		*p* > 0.999
	Bio Con vs. Bio ELS Female	*p* = 0.724	*P* < 0.0001^∗∗∗^	*p* = 0.018		*p* = 0.011
Effects of up-bringing	Male Bio Con vs. Con → Con	*p* = 0.387	*p* > 0.999	*p* = 0.668		*p* > 0.999
	Male Bio Con vs. Con → ELS	*p* = 0.0039	*p* > 0.999	*p* > 0.999		*p* > 0.999
	Male Bio ELS vs. ELS → ELS	*p* = 0.955	*p* = 0.311	*p* > 0.999		*p* > 0.999
	Male Bio ELS vs. ELS → Con	*p* > 0.999	*p* = 0.700	*p* = 0.998		*p* > 0.999
	Female Bio Con vs. Con → Con	*p* = 0.991	*p* = 0.011	*p* = 0.234		*p* = 0.999
	Female Bio Con vs. Con → ELS	*p* > 0.999	*p* = 0.026	*p* = 0.018		*p* = 0.947
	Female Bio ELS vs. ELS → ELS	*p* = 0.991	*p* = 0.996	*p* = 0.950		*p* > 0.999
	Female Bio ELS vs. ELS → Con	*p* = 0.992	*p* > 0.999	*p* = 0.978		*p* > 0.999
Effects of sex	Bio Con Male vs. Bio Con Female	*p* = 0.154	*p* = 0.027	*p* > 0.999		*p* = 0.0015
	Bio ELS Male vs. Bio ELS Female	*p* > 0.999	*p* = 0.700	*p* > 0.999		*p* > 0.999

*Bold: significant difference; ^∗∗∗^p < 0.0001. Shaded cells: Post hoc comparisons after interactions or main effects were found—corrected p-values shown.*

**Table 3:** ANOVA results for F2 Bndf methylation.

		bdnf				
	Comparison	CpG -35	CpG -23	CpG 19	CpG 35	CpG 43
Inter-actions	Lineage × Upbringing × Sex	*F*_2,24_ = 0.711; *p* = 0.501	*F*_2,24_ = 4.84; *p* = 0.02	*F*_2,24_ = 0.027; *p* = 0.973	*F*_2,24_ = 4.2; *p* = 0.0273	*F*_2,24_ = 0.77; *p* = 0.475
	Lineage × Upbringing	*F*_2,24_ = 2.02; *p* = 0.154	*F*_2,24_ = 5.08; *p* = 0.015	*F*_2,24_ = 0.189; *p* = 0.829	*F*_2,24_ = 8.76; *p* = 0.001	*F*_2,24_ = 3.12; *p* = 0.062
	Sex × Lineage	*F*_1,24_ = 3.12; *p* = 0.089	*F*_1,24_ = 3.76; *p* = 0.064	*F*_1,24_ = 0.004; *p* = 0.951	*F*_1,24_ = 7.00; *p* = 0.014	*F*_1,24_ = 7.14; *p* = 0.013
	Sex × Upbringing	*F*_2,24_ = 0.01; *p* = 0.992	*F*_2,24_ = 1.78; *p* = 0.190	*F*_2,24_ = 0.375; *p* = 0.692	*F*_2,24_ = 1.95; *p* = 0.164	*F*_2,24_ = 0.23; *p* = 0.795
Main effects	Lineage	*F*_1,24_ = 87.8^∗∗∗^	*F*_1,24_ = 3.76; *p* = 0.064	*F*_1,24_ = 0.652; *p* = 0.427	*F*_1,24_ = 72.74^∗∗∗^	*F*_1,24_ = 151.1^∗∗∗^
	Upbringing	*F*_2,24_ = 22.0 ^∗∗∗^	*F*_2,24_ = 2.36; *p* = 0.116	*F*_2,24_ = 2.33; *p* = 0.119	*F*_2,24_ = 0.2; *p* = 0.820	*F*_2,24_ = 2.30; *p* = 0.122
	Sex	*F*_1,24_ = 16.5; *p* = 0.0004	*F*_1,24_ = 15.1; *p* = 0.0007	*F*_1,24_ = 0.467; *p* = 0.501	*F*_1,24_ = 51.9^∗∗∗^	*F*_1,24_ = 0.29; *p* = 0.598
Effects of lineage	Bio Con vs. Bio ELS Male	*p* = 0.0007	*p* > 0.999		*p* = 0.0005	*p* < 0.0001^∗∗∗^
	Bio Con vs. Bio ELS Female	*p* = 0.006	*p* = 0.002		*p* = 0.0001	*p* = 0.005
Effects of up-bringing	Male Bio Con vs. Con → Con	*p* > 0.999	*p* > 0.999		*p* = 0.993	*p* = 0.879
	Male Bio Con vs. Con → ELS	*p* = 0.333	*p* = 0.999		*p* > 0.999	*p* = 0.507
	Male Bio ELS vs. ELS → ELS	*p* = 0.720	*p* > 0.999		*p* = 0.999	*p* = 0.711
	Male Bio ELS vs. ELS → Con	*p* = 0.068	*p* > 0.999		*p* = 0.993	*p* > 0.999
	Female Bio Con vs. Con → Con	*p* > 0.999	*p* = 0.996		*p* = 0.802	*p* > 0.999
	Female Bio Con vs. Con → ELS	*P* = 0.068	*p* > 0.999		*p* = 0.010	*p* > 0.999
	Female Bio ELS vs. ELS → ELS	*p* = 0.720	*p* = 0.014		*p* = 0.373	*p* = 0967
	Female Bio ELS vs. ELS → Con	*p* = 0.999	*p* = 0.009		*p* = 0.373	*p* > 0.999
Effects of sex	Bio Con Male vs. Bio Con Female	*p* = 0.586	*p* > 0.999		*p* = 0.477	*p* = 0.711
	Bio ELS Male vs. Bio ELS Female	*P* = 0.971	*p* = 0.001		*p* = 0.209	*p* = 0.880

*Bold: significant difference; ^∗∗∗^p < 0.0001. Shaded cells: Post hoc comparisons after interactions or main effects were found—corrected p-values shown.*

##### Nr3c1 methylation

**FIGURE 6:**
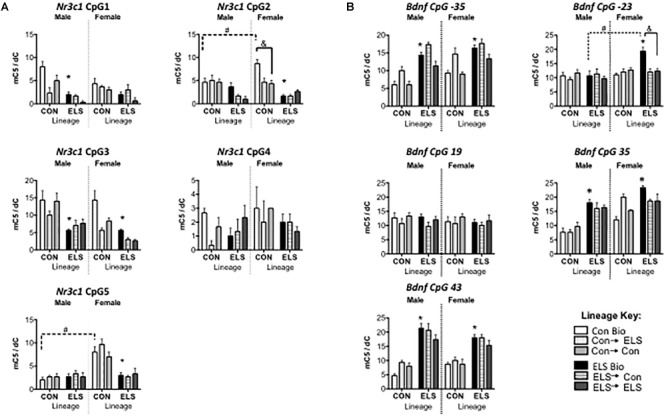
Methylation of hippocampal Nr3c1 **(A)** and Bdnf **(B)** in F2 generation males and females. Means ± SEM are shown. ^∗^*p* < 0.05 difference from Con Bio. ^&^*p* < 0.05 difference between upbringing groups within Con or ELS lineage. ^#^*p* < 0.05 difference between males and females of same lineage and upbringing. *n* = 3–4.

Three-way ANOVAs revealed significantly lower methylation in animals with ELS lineage (main effect of lineage) at CpG sites 1, 2, 3, and 5, main effects of upbringing at CpG sites 1, 2, and 3 and a main effect of sex at CpG sites 3 and 5, with fostered females showing lower methylation than males at site 3 (see Table 2), and with Con females showing higher methylation at site 5 (Figure 6A). A three-way interaction between sex, lineage, and upbringing was seen only at CpG site 2, which was driven by higher methylation in Con Bio females compared to all other groups (see Table 2).

##### Bdnf methylation

Three-way ANOVAs revealed higher methylation in animals with ELS lineage (main effect of lineage) at CpG sites -35, 35, and 43, a main effect of upbringing at CpG site -35, and higher methylation in females (main effect of sex) at CpG sites -35, -23, and 35 (Figure 6B). A three-way interaction between sex, lineage, and upbringing at site -23 was driven by higher methylation in ELS Bio females compared to all other groups (see Table 3). A three-way interaction at site 35 was driven by the fact that fostering Con females to ELS dams (Con → ELS) raised methylation levels to ELS Bio levels, while fostering had no effect in males.

#### F2 Gene Expression

##### Nr3c1 expression

**FIGURE 7:**
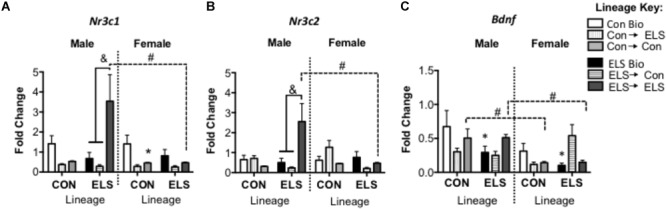
Hippocampal mRNA expression of Nr3c1 **(A)**, Nr3c2 **(B)**, and Bdnf **(C)** in F2 generation males and females. Means ± SEM are shown. ^∗^*p* < 0.05 difference from Con Bio (^∗^ over male and female ELS Bio represents a main effect of lineage, rather than a pairwise comparison). ^&^*p* < 0.05 difference between upbringing groups within Con or ELS lineage. ^#^*p* < 0.05 difference between males and females of same lineage and upbringing. *n* = 6–8.

A three-way ANOVA revealed significant main effects of sex (*F*_1,82_ = 4.481; *p* = 0.038) and upbringing (*F*_2,82_ = 5.888; *p* = 0.004), as well as significant interactions of sex × lineage × upbringing (*F*_2,82_ = 3.719, *p* = 0.029), lineage × upbringing (*F*_2,82_ = 6.047, *p* = 0.004), and sex × upbringing (*F*_2,82_ = 3.872, *p* = 0.025) (Figure 7A). *Post hoc* analyses revealed that in non-fostered males and females, lineage alone did not affect Nr3c1 expression. However, Con-born females fostered to Con mothers (Con → Con; *p* = 0.045) had lower expression when compared to those not fostered (Con Bio). This effect was not observed in females with ELS lineage. However, ELS-lineage males who were fostered to ELS dams (ELS → ELS males) had higher expression of Nr3c1 compared to those that were not fostered (ELS Bio) (*p* = 0.0014) and compared to those who were fostered to Con dams (ELS → Con) (*p* = 0.0003). Finally, ELS → ELS males had higher expression compared to their female counterparts (*p* = 0.0031).

##### Nr3c2 expression

A three-way ANOVA revealed significant interactions of sex × lineage × upbringing (*F*_2,80_ = 3.538, *p* = 0.034), sex × lineage (*F*_1,80_ = 5.231; *p* = 0.025), sex × upbringing (*F*_2,80_ = 3.947, *p* = 0.024), and lineage × upbringing (*F*_2,80_ = 8.604, *p* < 0.001) (Figure 7B). *Post hoc* analyses revealed that in males, Nr3c2 expression followed a similar pattern to Nr3c1, with ELS → ELS males expressing more Nr3c2 compared to both ELS → Con males (*p* = 0.0002) and ELS Bio males (*p* = 0.0007). Again, these ELS → ELS males show higher expression compared to their female counterparts (*p* = 0.036).

##### Bdnf expression

A three-way ANOVA revealed a significant main effect of sex (*F*_1,79_ = 8.335; *p* = 0.005), with females expressing overall lower levels compared to males, as well as significant interactions of lineage × upbringing (*F*_2,79_ = 5.010; *p* = 0.009) and sex × upbringing (*F*_2,79_ = 3.860; *p* = 0.026) (Figure 7C). Sex differences were revealed in groups fostered within condition, as Con → Con males and ELS → ELS males showed higher Bdnf expression when compared to their female counterparts (*p* = 0.0048 and *p* = 0.0138, respectively). In order to uncover effects of lineage alone, subsequent two-way ANOVA (Sex × Lineage) in subjects that were not fostered revealed main effects of lineage (*F*_1,23_ = 5.204; *p* = 0.032) and sex (*F*_1,23_ = 4.515; *p* = 0.045); ELS Bio subjects expressed lower levels of Bdnf than Con Bio, and females expressed less Bdnf than males. Pair-wise comparisons did not reveal any differences in *post hoc* tests.

#### F2 Open Field Behavior

##### General locomotion

**FIGURE 8:**
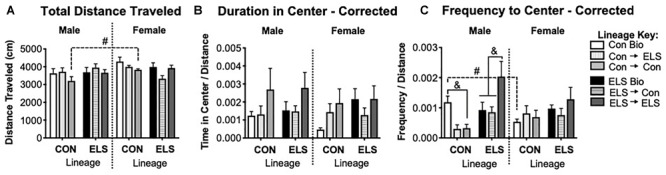
Distance traveled **(A)**, duration of time spent in the center **(B)**, and frequency of visits to the center **(C)** of an open field in F2 generation males and females. Means ± SEM are shown. ^&^*p* < 0.05 difference between upbringing groups within Con or ELS lineage. ^#^*p* < 0.05 difference between males and females of same lineage and upbringing. *n* = 6–8.

A three-way ANOVA revealed a trending main effect of sex (*F*_1,80_ = 3.949; *p* = 0.051) and a significant interaction of sex × lineage (*F*_1,80_ = 4.513; *p* = 0.037) on total distance traveled in the OFT (Figure 8A). *Post hoc* analyses revealed that the sex difference was driven by males with Con lineage fostered to Con dams (Con → Con) traveling significantly less than their female counterparts (*p* = 0.0345). Subsequent two-way ANOVAs revealed an additional significant main effect of lineage for females (*F*_1,30_ = 4.214; *p* = 0.0482), as well as a significant main effect of upbringing (*F*_2,30_ = 3.87; *p* = 0.032) in females. Since lineage and upbringing appeared to affect general locomotion, the rest of OFT behaviors were normalized to distance traveled (see section “Materials and Methods”), with uncorrected data shown in Supplementary Figure S3.

##### Center time and visits

A three-way ANOVA revealed no significant main effects or interactions on duration of time in the center of the open field (Figure 8B). A significant interaction of sex × lineage × upbringing (*F*_2,75_ = 3.208; *p* = 0.047) was found for frequency of visits to the center (Figure 8C), as well as a trending main effect of upbringing in males (*F*_2,36_ = 3.142, *p* = 0.0552). *Post hoc* analyses confirmed that Con → Con males made fewer visits to the center than Con Bio males (*p* = 0.04), Con → ELS males were not different from Con → Con but were also not significantly different from Con Bio (*p* = 0.08), and ELS → ELS males visited the center more often than ELS Bio (*p* = 0.016) and ELS → Con (*p* = 0.005). Additionally, non-fostered Con Bio males showed significantly greater frequency to center compared to their female counterparts (*p* = 0.0407).

#### F2 Corticosterone Secretion

A three-way repeated measures ANOVA revealed a significant main effect of time (*F*_1,68_ = 251.773; *p* < 0.001), showing that exposure to a novel open field increased CORT secretion.

**FIGURE 9:**
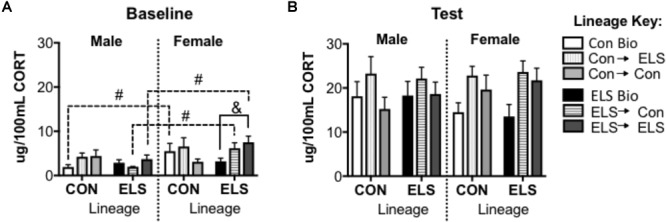
Serum corticosterone at baseline **(A)** and post-exposure to an open field arena and a conspecific **(B)** in F2 generation males and females. ^&^*p* < 0.05 difference between upbringing groups within Con or ELS lineage. ^#^*p* < 0.05 difference between males and females of same lineage and upbringing. *n* = 6–8.

For baseline CORT, a subsequent three-way ANOVA revealed an additional significant main effect of sex (*F*_1,68_ = 10.195; *p* = 0.002), and a significant interaction of sex × lineage × upbringing (*F*_2,68_ = 3.214; *p* = 0.048) (Figure 9A). *Post hoc* analyses revealed that Con Bio females expressed higher baseline CORT levels that Con Bio males (*p* = 0.0081), but this sex difference was not observed in ELS Bio animals. Fostering of females with ELS lineage, however, brought CORT levels up to resemble Con Bio females, yielding higher levels than their male counterparts (male vs. female ELS → Con *p* = 0.02; male vs. female ELS → ELS *p* = 0.014). Additionally, females of ELS lineage fostered to ELS dams (ELS → ELS females) had significantly higher baseline CORT than ELS Bio females (*p* = 0.0117). CORT levels following exposure to a novel environment were not different between groups (Figure 9B).

### Impact of Estrous Phase

For the F1 generation, on the day of behavior and tissue collection, 31% of females were in proestrus, 12.5% were in estrus, 18.8% were in metestrus, and 37.5% were in diestrus, dispersed throughout the groups (see Supplementary Table S2A). No significant effects of estrous phase were found for corticosterone levels or behavior (data not shown). For the F2 generation, on the day of behavior and tissue collection 33% of females were in proestrus, 39.2% were in estrus, 10.8% were in metestrus, and 16.4% were in diestrus, dispersed throughout the groups (see Supplementary Table S2B). Again, no significant effects of estrous phase were found for corticosterone levels or behavior (data not shown).

## Discussion

### Direct Effects of Maternal Separation

**FIGURE 10:**
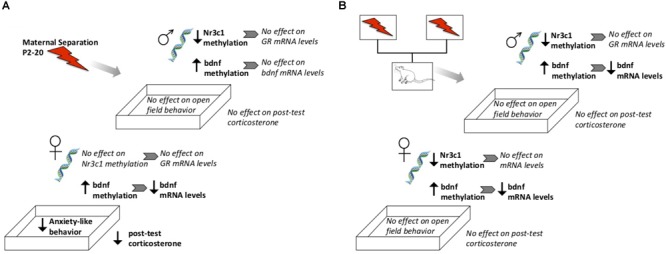
Schematic diagram highlighting general observed effects after direct exposure to ELS **(A)** or from genetic lineage of ELS-exposed parents **(B)**. Not shown here are direct effects of fostering to a different parent, or baseline sex differences.

We report that ELS exposure had sex-specific effects on gene methylation, gene expression, and HPA function in adulthood (see Figure 10 for a summary). As previously reported in studies using similar ELS procedures, maternal separation resulted in higher methylation and lower expression of Bdnf exons in adulthood (Seo et al., 2016; Dandi et al., 2018). Interestingly, we observed effects on gene expression mostly in females. While sex-specific assessments of Bdnf methylation or expression following ELS are scarce, similar female-specific effects of ELS on Bdnf epigenetic regulation have also been found in limited-bedding paradigms (Doherty et al., 2016). Importantly, our data speak to the fact that altered methylation does not always translate to altered gene expression. While tissue was taken from the same animals, even though ELS led to lower methylation of the Nr3c1 gene in males, ELS-exposed males did not express altered levels of Nr3c1 mRNA. Since we did not measure protein levels of GRs or BDNF, we cannot determine whether translation was affected in these animals; however, previous studies have reported that changes in the BDNF methylation status does result in downstream changes in BDNF protein levels (Trivedi and Deth, 2014). Such methylation status includes the methylation of mRNA and protein that can affect the translation machinery and result in altered protein levels. Such roles of mRNA were not explored in the current study but have been implicated in neurodevelopmental disorders such as autism spectrum disorder (Liyanage, 2016).

With regards to behavior, ELS-exposed subjects were more active than control subjects upon introduction to the open field arena, which may reflect a higher response to novelty that we have previously reported (Ganguly et al., 2015). Surprisingly, while ELS has been repeatedly shown to increase anxiety-like behaviors (Koe et al., 2016), here we observed that even after correcting for increased general locomotion, females exposed to ELS visited the center of the open field more often than control subjects—typically a measure of lower anxiety. One explanation for this discrepancy is that our subjects were handled immediately prior to the open field test. Given that handling is a mild stressor and ELS can reduce some effects of subsequent stressors (Hsiao et al., 2016), it is possible that anxiety-like behaviors were increased by handling in our control groups but not the ELS groups. It is important to note here that assays such as the OFT have limited validity in recapitulating human anxiety, and increased visits to the center may be interpreted as ELS-induced alterations in decision-making, risk-taking, or other threat-appraisal behaviors.

Additionally, exposure to the open field arena followed by exposure to a conspecific yielded a lower CORT response in ELS-exposed females compared to Con females, whereas ELS had no effect on baseline CORT. Therefore, increased locomotion and visits to the center of an open field were associated with blunted CORT response in ELS-exposed females. Blunted HPA responsivity has also been reported in humans following childhood institutionalization (McLaughlin et al., 2015), and in animal models of ELS (Biggio et al., 2014; Fuentes et al., 2018; Gehrand et al., 2018) (but see Parfitt et al., 2007 for a distinction in mice).

As hypothesized, early life stress alters methylation levels of stress-associated genes (and in some cases expression levels), and blunts stress-responses in a sex-dependent manner. In contrast to our hypotheses, stressed females did not show increased anxiety-like behaviors in the OFT, instead showing increased visits to the center. We suggest that this may be a result of increased stimulus-seeking, but future studies will require combining multiple anxiety assays to gain behavioral resolution with regards to the effects of early life stress.

### Does a History of Early Life Stress Impact Later Dam-Pup Interactions?

Previous exposure to ELS affected the nursing behavior of dams caring for neonates (PD6–7). While not all maternal behaviors were altered by early life experiences alone, ELS-exposed dams spent more of their time nursing than Con-exposed dams, and this difference was driven by longer bouts in a passive posture. ELS-exposed dams also made shorter excursions away from the nest compared to Con dams. This alteration of maternal behavior by earlier exposure to stress in infancy has been previously reported (Murgatroyd and Nephew, 2013), and has been associated with epigenetic programming of the HPA axis (Murgatroyd and Spengler, 2011). Interestingly, ELS exposure was not the only factor that influenced maternal behavior; the genetic lineage of the litter also affected nursing. While fostering itself did not affect the length of passive nursing bouts, raising pups born to a Con dam but not pups born to a different ELS dam normalized atypical maternal behavior in ELS dams. Relatedly, when Con dams raised pups with ELS lineage, dams tended to leave the nest more often and spend less time away after each exit. This behavior is reminiscent of behaviors exhibited by dams currently undergoing an impoverished caregiving or separation paradigm (Walker et al., 2017); in other words, pups whose biological mothers experienced ELS appear to impart that behavior on their caregiver. It is therefore possible that the behaviors of pups with Con lineage, such as vocalizations, rooting behaviors, or potentially even microbiota-associated odors, were different from those of pups with ELS lineage, which influenced the behavior of the dam. This hypothesis will require further investigation. Regardless of the underlying mechanism, it is clear that the lineage and the upbringing of offspring can interact to influence the developmental environment.

### Does Parental Exposure to Early Life Stress Affect Offspring’s DNA Methylation and Gene Expression of Glucocorticoid Receptors and Bdnf?

Epigenetic changes induced by ELS were transmitted cross-generationally; subjects with ELS lineage expressed higher methylation and lower gene expression levels of Bdnf, similar to their parent’s generation (see Figure 10 for a summary). Genetic lineage from ELS or Con parents alone did not affect hippocampal expression of Nr3c1 or Nr3c2. Again, gene expression did not consistently correspond to observed methylation levels, since genetic lineage from ELS-exposed parents did lead to cross-generational lower methylation of Nr3c1 in adult offspring. Fostering also had epigenetic effects on Nr3c1 and Bdnf that did not translate to gene expression changes. In other words, early life caregiving from a dam previously exposed to ELS alters epigenetic regulation of Bdnf and Nr3c1. As hypothesized, animals descended from ELS parents showed alterations in gene methylation and expression, but upbringing effects were not as pronounced, and not always in the direction that we expected (i.e., Con upbringing did not always ameliorate and ELS upbringing did not always compound observed lineage effects). Previous reports of decreased histone acetylation of prefrontal cortex Bdnf promoters in maltreated pups (Blaze et al., 2015) and epigenetic alterations in hippocampus Nr3c1 in pups exposed to high maternal care mothers (McGowan et al., 2011) support the hypothesis that altered maternal behavior by ELS-exposed dams was responsible for the changes we observed.

### Does Parental Exposure to Early Life Stress Affect Offspring’s HPA Function or Anxiety-Like Behavior?

There was a sex difference in baseline CORT, with higher levels in females as have been previously reported (Spencer and Deak, 2017). While a lack of difference between Con Bio and ELS Bio implies that lineage did not appear to affect CORT levels, the sex difference was lost in animals with ELS lineage. In other words, baseline CORT was reduced in females born to ELS-exposed parents to levels that more closely resembled both Con Bio and ELS Bio males. Since HPA responsivity to a mild stressor was also blunted in F1 females exposed to ELS, it appears that this phenotype was transferred to female offspring. This inherited effect of ELS is consistent with our hypotheses. Notably, *trans*-generational effects of ELS via a daily male intruder during lactation, with consequential reduced maternal care (Nephew and Bridges, 2011; Babb et al., 2014) also reduces CORT in the F2 generation, though effects were seen in juvenility but not adulthood. In contrast to our hypotheses, genetic lineage alone was not found to affect anxiety-like behavior in the OFT (Figure 10). Interestingly – and unexpectedly, based on our hypotheses and F1 findings – males with ELS lineage who were fostered to ELS dams showed a dramatic increase in visits to center. This is in contrast to OFT results in F1, where ELS females showed a similar increase. It is unclear what exactly is underlying this effect, but the findings suggest that females may be more behaviorally sensitive to direct stressors (i.e., F1), whereas males may be more so to second-hand stressors (i.e., F2). It is important to highlight that the maternal separation ELS paradigm investigated here is one of several types of adversity models, each of which could have separate lifetime and cross-generational effects. Additionally, while we have no knowledge of any differences in lineage prior to the onset of the study, outcome measures could always be further mediated by lineage effects that exist within any particular strain of rats.

### Effects of Fostering

Counter to our original hypothesis, subjects’ upbringing was more influential to some of our measures of interest — such as HPA regulators and anxiety-like behavior — than genetic lineage. For example, fostering to a different mother yielded baseline CORT levels in ELS-lineage females that were similar to levels in Con-lineage females. While fostering females appeared to impact CORT, fostering Con males to a different mother provoked anxiety-like behavior in adulthood. In males with ELS lineage, however, fostering to an ELS-exposed dam appeared to provoke the opposite behavior in adulthood, with more time spent in the center of an open field. In several cases, being raised by an ELS-exposed dam specifically, rather than fostering itself, also affected Nr3c1 and Nr3c2 gene expression outcomes. While these studies were not sufficiently powered to investigate correlations between maternal behavior and subsequent measures in the offspring, it is possible that differential maternal behavior received by fostered pups drove changes in gene expression, HPA activity, and behavior. As noted above, the lineage of the pups themselves may have influenced their own early environment via their impact on maternal behavior.

## Conclusion

Taken together, the data presented here suggest that maternal separation ELS generates an altered epigenetic lineage, which importantly interacts with the caretaking environment to affect gene expression, HPA responsiveness, and behavior. A surprising finding indicates that not only the mother’s history, but also the interplay between a mother’s experience and the litter’s genetic lineage, influences gene expression and stress responsivity in the offspring. It is important to note that the current study cannot decipher whether early life stress *per se*, rather than stress exposure at any phase of life, can lead to inherited epigenetic or caretaking-induced changes in future generations. This work leads to further questions regarding lineage effects on infant behavior, effects of these manipulations on protein translation, and wider cross-generational influences on behavior.

## Author Contributions

EC led experimental design, conducted all breeding procedures, brain dissections, PCR analyses, data analyses, and wrote several parts of the manuscript. PG helped design and conduct all PCR analyses. JH conducted corticosterone ELISAs and helped with manuscript preparation. SP conducted most of the behavioral procedures. CD conducted maternal behavior recording, conducted much of the data analyses, and wrote several parts of the manuscript. NR, NA, MH, and MT conducted all bisulfite sequencing procedures and helped with manuscript preparation. HB oversaw all experimental design, procedures, analyses, and manuscript preparation, and conducted several parts of experimentation, analyses, and manuscript preparation.

## Conflict of Interest Statement

The authors declare that the research was conducted in the absence of any commercial or financial relationships that could be construed as a potential conflict of interest.

## Footnotes

1 MyAssays.com
