# Supplementary material for: Cross-Generational Transmission of Early Life Stress Effects on HPA Regulators and Bdnf Are Mediated by Sex, Lineage, and Upbringing
Source: Front Behav Neurosci. 2019 May 9;13:101. doi: 10.3389/fnbeh.2019.00101 (PMC6521572; doi:10.3389/fnbeh.2019.00101)
Supplement: Supplementary file 1 [file Table_1.doc]

| **A. P6- 7** | **Bio** | | | **Fostered Across Condition** | | | **Fostered Within Condition** | | |  |  |
| --- | --- | --- | --- | --- | --- | --- | --- | --- | --- | --- | --- |
| **Lineage** | **Mean** |  | **SEM** | **Mean** |  | **SEM** | **Mean** |  | **SEM** |  |  |
| Con | 1087.8 | ± | 299.0 | 1890.3 | ± | 528.9 | 2285.5 | ± | 265.4 | **Seconds On Nest** | |
| ELS | 2278.3 | ± | 50.4 | 1638.5 | ± | 112.8 | 1931.5 | ± | 652.4 |  |  |
|  |  |  |  |  |  |  |  |  |  |  |  |
| Con | 2.3 | ± | 0.9 | 5.3 | ± | 1.2 | 5.0 | ± | 0.0 | **# Bouts On Nest** | |
| ELS | 4.0 | ± | 0.6 | 6.0 | ± | 0.6 | 6.0 | ± | 0.0 |  |  |
|  |  |  |  |  |  |  |  |  |  |  |  |
| Con | 591.9 | ± | 191.2 | 342.3 | ± | 27.0 | 387.9 | ± | 30.4 | **Seconds/Bout On Nest** | |
| ELS | 598.0 | ± | 101.0 | 280.7 | ± | 43.9 | 356.8 | ± | 73.9 |  |  |
|  |  |  |  |  |  |  |  |  |  |  |  |
| Con | 206.2 | ± | 92.2 | 676.1 | ± | 426.4 | 497.3 | ± | 183.0 | **Seconds Arched Nursing** | |
| ELS | 213.4 | ± | 114.8 | 806.3 | ± | 237.7 | 548.2 | ± | 284.9 |  |  |
|  |  |  |  |  |  |  |  |  |  |  |  |
| Con | 2.0 | ± | 0.6 | 5.0 | ± | 0.0 | 3.7 | ± | 0.9 | **# Bouts of Arched Nursing** | |
| ELS | 1.7 | ± | 0.9 | 5.3 | ± | 0.9 | 2.7 | ± | 1.5 |  |  |
|  |  |  |  |  |  |  |  |  |  |  |  |
| Con | 91.1 | ± | 38.7 | 202.8 | ± | 90.0 | 180.8 | ± | 94.4 | **Seconds/Bout Arched Nursing** | |
| ELS | 139.5 | ± | 57.2 | 146.2 | ± | 19.7 | 228.3 | ± | 90.7 |  |  |
|  |  |  |  |  |  |  |  |  |  |  |  |
| Con | 126.1 | ± | 50.1 | 185.8 | ± | 27.3 | 148.5 | ± | 64.8 | **Seconds Licking/Grooming** | |
| ELS | 129.4 | ± | 50.4 | 119.7 | ± | 59.5 | 148.1 | ± | 113.7 |  |  |
|  |  |  |  |  |  |  |  |  |  |  |  |
| Con | 10.3 | ± | 3.2 | 15.0 | ± | 0.6 | 11.3 | ± | 2.4 | **# Bouts Licking/Grooming** | |
| ELS | 10.3 | ± | 1.9 | 11.3 | ± | 6.8 | 15.0 | ± | 10.7 |  |  |
|  |  |  |  |  |  |  |  |  |  |  |  |
| Con | 10.9 | ± | 2.1 | 12.3 | ± | 1.6 | 12.3 | ± | 3.1 | **Seconds/Bout Licking/Grooming** | |
| ELS | 11.7 |  | 2.4 | 12.2 |  | 1.7 | 7.0 |  | 2.6 |  |  |
|  |  |  |  |  |  |  |  |  |  |  |  |

Supplemental Table 1: Maternal behavior measures across four sampling timepoints

| **B. P13-14** | **Bio** | | | **Fostered Across Condition** | | | **Fostered Within Condition** | | |  |  |
| --- | --- | --- | --- | --- | --- | --- | --- | --- | --- | --- | --- |
| **Lineage** | **Mean** |  | **SEM** | **Mean** |  | **SEM** | **Mean** |  | **SEM** |  |  |
| Con | 1484.4 | ± | 258.7 | 1988.3 | ± | 706.2 | 2698.7 | ± | 193.1 | **Seconds Off Nest** | |
| ELS | 1671.5 | ± | 392.1 | 2522.6 | ± | 632.7 | 1820.6 | ± | 491.7 |  |  |
| Con | 5.0 | ± | 0.6 | 7.3 | ± | 2.4 | 5.7 | ± | 1.2 | **# Bouts Off Nest** | |
| ELS | 5.3 | ± | 0.9 | 4.3 | ± | 1.5 | 9.0 | ± | 0.0 |  |  |
| Con | 251.4 | ± | 256.2 | 840.0 | ± | 132.9 | 754.5 | ± | 340.9 | **Seconds/Bout Off Nest** | |
| ELS | 476.3 | ± | 229.3 | 472.7 | ± | 635.5 | 477.9 | ± | 310.8 |  |  |
| Con | 1875.6 | ± | 258.7 | 1371.7 | ± | 706.2 | 661.3 | ± | 193.1 | **Seconds On Nest** | |
| ELS | 1678.0 | ± | 383.5 | 837.4 | ± | 632.7 | 1539.4 | ± | 491.7 |  |  |
| Con | 3.7 | ± | 0.9 | 5.7 | ± | 3.5 | 3.0 | ± | 0.0 | **# Bouts On Nest** | |
| ELS | 5.3 | ± | 1.2 | 3.0 | ± | 0.0 | 6.0 | ± | 0.6 |  |  |
| Con | 550.0 | ± | 85.0 | 308.5 | ± | 161.3 | 192.3 | ± | 72.1 | **Seconds/Bout On Nest** | |
| ELS | 315.8 | ± | 11.7 | 279.1 | ± | 210.9 | 261.1 | ± | 86.8 |  |  |
| Con | 688.3 | ± | 54.6 | 333.2 | ± | 209.6 | 106.8 | ± | 58.3 | **Seconds Arched Nursing** | |
| ELS | 643.8 | ± | 193.2 | 670.3 | ± | 590.5 | 102.0 | ± | 71.6 |  |  |
| Con | 2.7 | ± | 0.9 | 2.7 | ± | 2.2 | 1.0 | ± | 0.6 | **# Bouts of Arched Nursing** | |
| ELS | 3.0 | ± | 0.6 | 1.0 | ± | 0.0 | 1.0 | ± | 0.6 |  |  |
| Con | 337.8 | ± | 122.4 | 380.0 | ± | 340.0 | 110.1 | ± | 9.9 | **Seconds/Bout Arched Nursing** | |
| ELS | 245.7 | ± | 88.0 | 207.9 | ± | 130.6 | 136.5 | ± | 103.5 |  |  |
| Con | 1229.2 | ± | 263.6 | 1182.2 | ± | 363.3 | 878.2 | ± | 311.4 | **Seconds Passive Nursing** | |
| ELS | 1056.1 | ± | 508.2 | 768.8 | ± | 293.5 | 1329.1 | ± | 386.0 |  |  |
| Con | 5.0 | ± | 1.2 | 6.3 | ± | 3.9 | 4.0 | ± | 0.0 | **# Bouts Passive Nursing** | |
| ELS | 5.7 | ± | 2.0 | 3.7 | ± | 0.9 | 5.0 | ± | 0.0 |  |  |
| Con | 247.7 | ± | 19.7 | 315.8 | ± | 104.1 | 339.5 | ± | 193.2 | **Seconds/Bout Passive Nursing** | |
| ELS | 158.1 | ± | 40.5 | 189.0 | ± | 47.2 | 433.8 | ± | 214.4 |  |  |
| Con | 172.3 | ± | 96.2 | 140.7 | ± | 123.6 | 100.9 | ± | 54.4 | **Seconds Licking/Grooming** | |
| ELS | 380.8 | ± | 140.5 | 57.4 | ± | 31.7 | 193.6 | ± | 96.8 |  |  |
| Con | 18.0 | ± | 10.4 | 13.3 | ± | 11.4 | 14.0 | ± | 7.6 | **# Bouts Licking/Grooming** | |
| ELS | 26.3 | ± | 13.0 | 6.0 | ± | 2.9 | 16.3 | ± | 8.4 |  |  |
| Con | 9.7 | ± | 0.5 | 9.8 | ± | 1.0 | 7.2 | ± | 0.0 | **Seconds/Bout Licking/Grooming** | |
| ELS | 17.9 | ± | 6.0 | 13.0 | ± | 5.5 | 12.1 | ± | 1.9 |  |  |
