# Supplementary material for: Cross-Generational Transmission of Early Life Stress Effects on HPA Regulators and Bdnf Are Mediated by Sex, Lineage, and Upbringing
Source: Front Behav Neurosci. 2019 May 9;13:101. doi: 10.3389/fnbeh.2019.00101 (PMC6521572; doi:10.3389/fnbeh.2019.00101)
Supplement: Supplementary file 2 [file Table_2.DOCX]

**Supplemental Table 2A: F1 generation estrous phase distributions**

|  | **Proestrus** | **Estrus** | **Metestrus** | **Diestrus** |
| --- | --- | --- | --- | --- |
| Con | 50% | 12.5% | 25% | 12.5% |
| ELS | 12.5% | 12.5% | 12.5% | 62.5% |

**Supplemental Table 2B: F2 generation estrous phase distributions**

|  | **Proestrus** | **Estrus** | **Metestrus** | **Diestrus** |
| --- | --- | --- | --- | --- |
| Con Bio | 33% | 50% | 0% | 16% |
| Con🡪ELS | 50% | 16% | 16% | 16% |
| Con🡪Con | 16% | 67% | 16% | 0% |
| ELS Bio | 16% | 50% | 16% | 16% |
| ELS🡪Con | 50% | 16% | 0% | 33% |
| ELS🡪ELS | 33% | 33% | 16% | 16% |
