# Supplementary material for: Cross-Generational Transmission of Early Life Stress Effects on HPA Regulators and Bdnf Are Mediated by Sex, Lineage, and Upbringing
Source: Front Behav Neurosci. 2019 May 9;13:101. doi: 10.3389/fnbeh.2019.00101 (PMC6521572; doi:10.3389/fnbeh.2019.00101)
Supplement: Supplementary file 3 [file Presentation_1.pptx]

## Slide 1
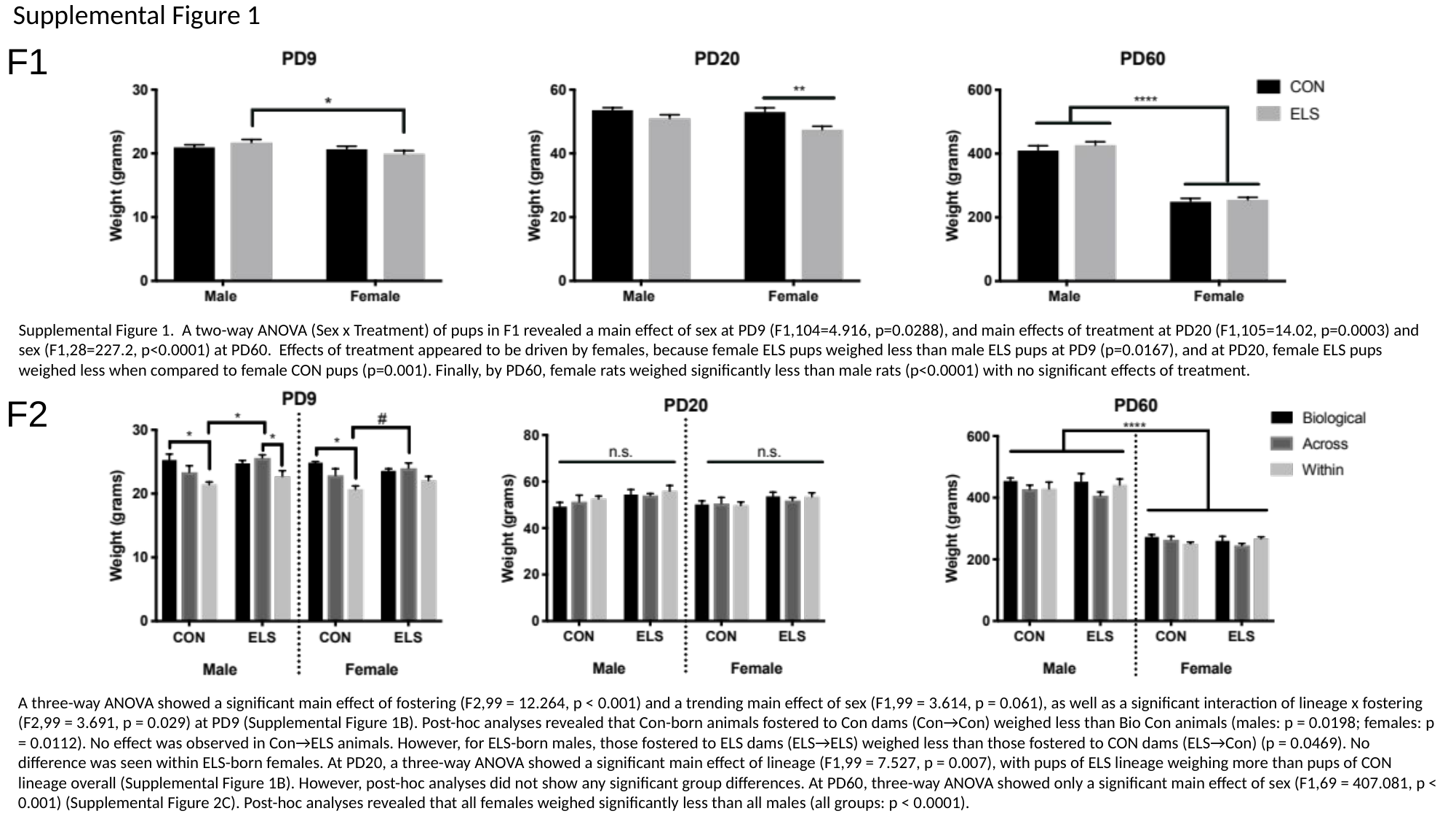

Supplemental Figure 1
F1
Supplemental Figure 1. A two-way ANOVA (Sex x Treatment) of pups in F1 revealed a main effect of sex at PD9 (F1,104=4.916, p=0.0288), and main effects of treatment at PD20 (F1,105=14.02, p=0.0003) and sex (F1,28=227.2, p<0.0001) at PD60. Effects of treatment appeared to be driven by females, because female ELS pups weighed less than male ELS pups at PD9 (p=0.0167), and at PD20, female ELS pups weighed less when compared to female CON pups (p=0.001). Finally, by PD60, female rats weighed significantly less than male rats (p<0.0001) with no significant effects of treatment.
F2
A three-way ANOVA showed a significant main effect of fostering (F2,99 = 12.264, p < 0.001) and a trending main effect of sex (F1,99 = 3.614, p = 0.061), as well as a significant interaction of lineage x fostering (F2,99 = 3.691, p = 0.029) at PD9 (Supplemental Figure 1B). Post-hoc analyses revealed that Con-born animals fostered to Con dams (Con→Con) weighed less than Bio Con animals (males: p = 0.0198; females: p = 0.0112). No effect was observed in Con→ELS animals. However, for ELS-born males, those fostered to ELS dams (ELS→ELS) weighed less than those fostered to CON dams (ELS→Con) (p = 0.0469). No difference was seen within ELS-born females. At PD20, a three-way ANOVA showed a significant main effect of lineage (F1,99 = 7.527, p = 0.007), with pups of ELS lineage weighing more than pups of CON lineage overall (Supplemental Figure 1B). However, post-hoc analyses did not show any significant group differences. At PD60, three-way ANOVA showed only a significant main effect of sex (F1,69 = 407.081, p < 0.001) (Supplemental Figure 2C). Post-hoc analyses revealed that all females weighed significantly less than all males (all groups: p < 0.0001).

## Slide 2
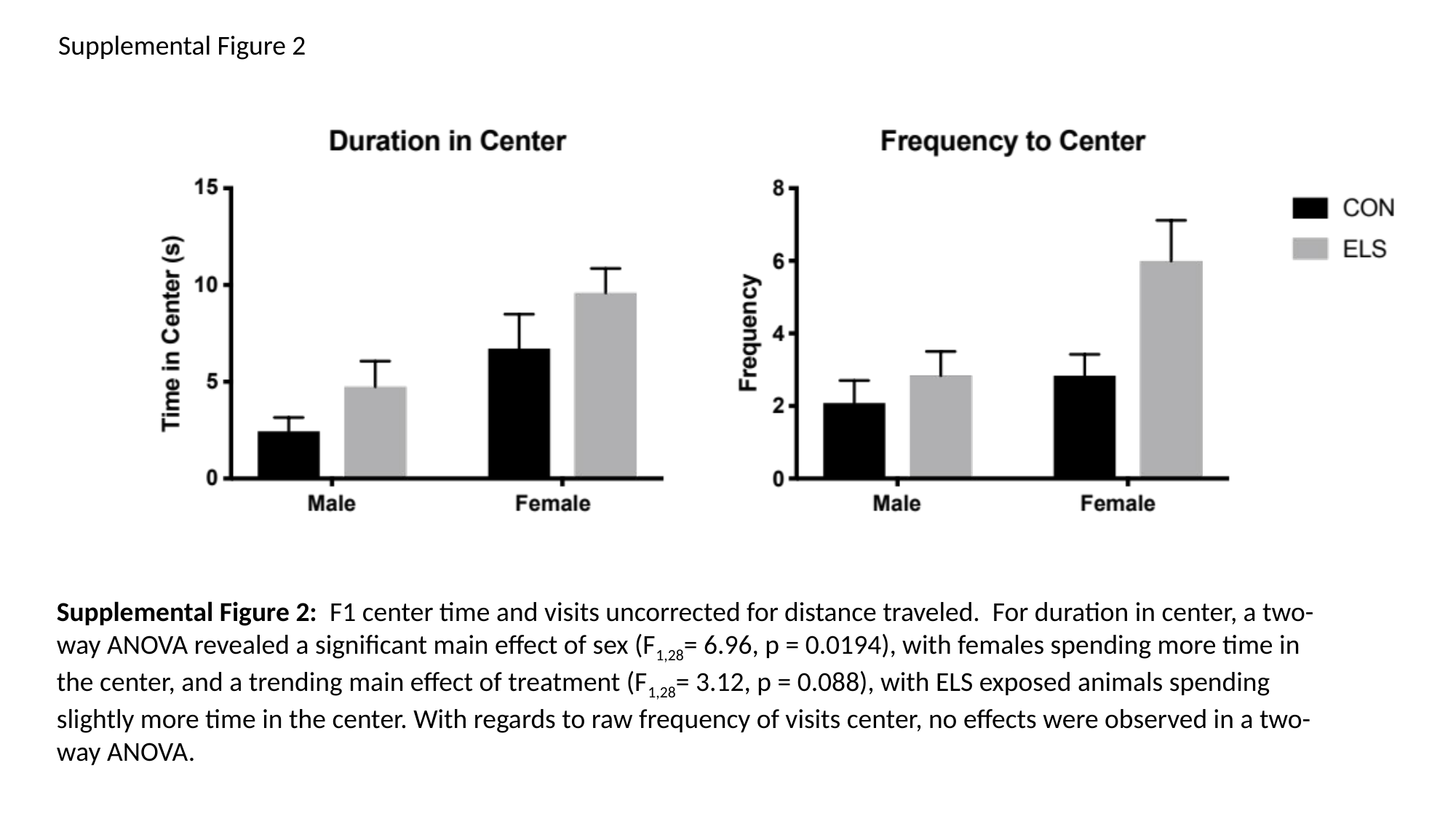

Supplemental Figure 2
Supplemental Figure 2: F1 center time and visits uncorrected for distance traveled. For duration in center, a two-way ANOVA revealed a significant main effect of sex (F1,28= 6.96, p = 0.0194), with females spending more time in the center, and a trending main effect of treatment (F1,28= 3.12, p = 0.088), with ELS exposed animals spending slightly more time in the center. With regards to raw frequency of visits center, no effects were observed in a two-way ANOVA.

## Slide 3
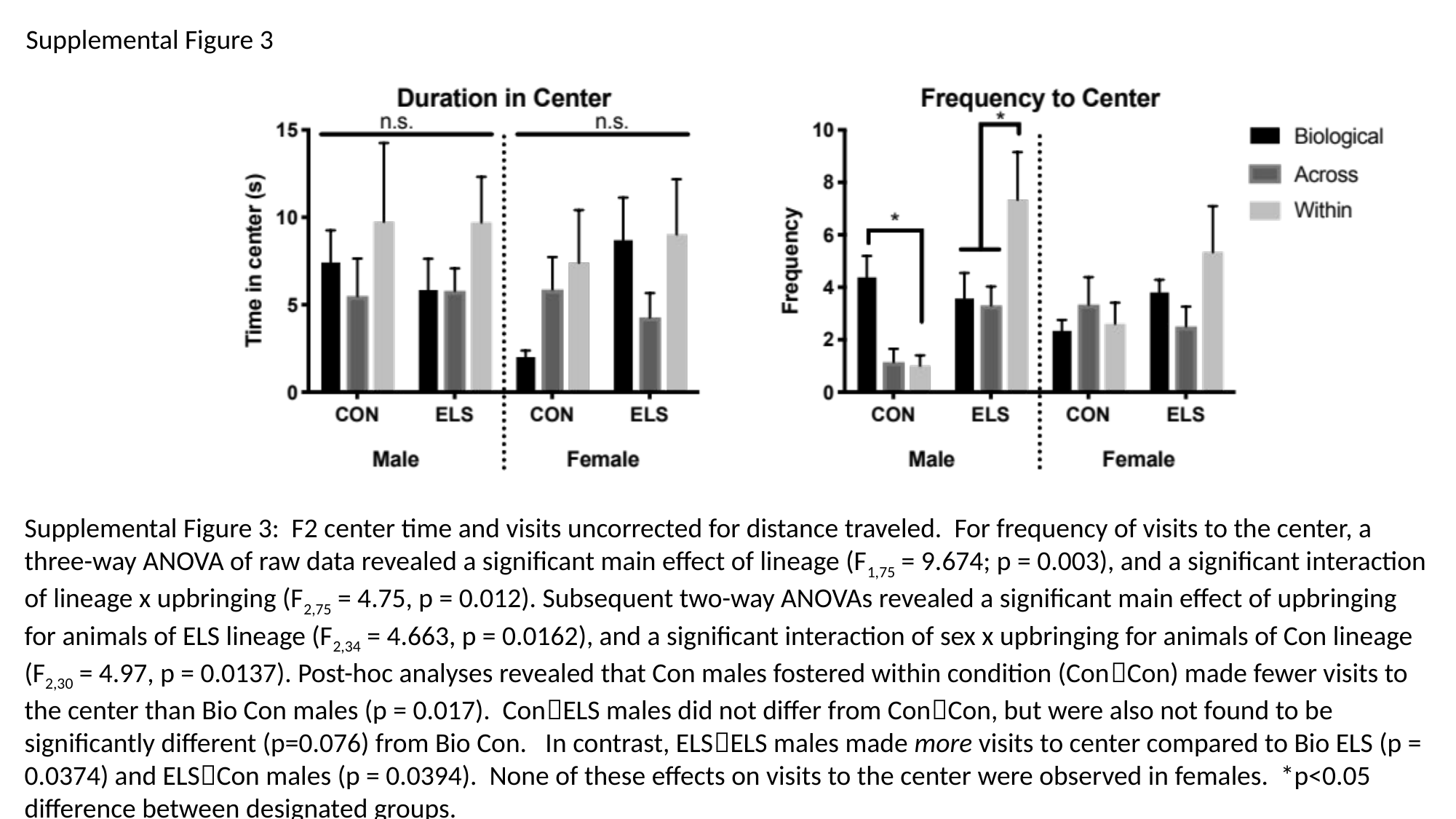

Supplemental Figure 3
Supplemental Figure 3: F2 center time and visits uncorrected for distance traveled. For frequency of visits to the center, a three-way ANOVA of raw data revealed a significant main effect of lineage (F1,75 = 9.674; p = 0.003), and a significant interaction of lineage x upbringing (F2,75 = 4.75, p = 0.012). Subsequent two-way ANOVAs revealed a significant main effect of upbringing for animals of ELS lineage (F2,34 = 4.663, p = 0.0162), and a significant interaction of sex x upbringing for animals of Con lineage (F2,30 = 4.97, p = 0.0137). Post-hoc analyses revealed that Con males fostered within condition (ConCon) made fewer visits to the center than Bio Con males (p = 0.017). ConELS males did not differ from ConCon, but were also not found to be significantly different (p=0.076) from Bio Con. In contrast, ELSELS males made more visits to center compared to Bio ELS (p = 0.0374) and ELSCon males (p = 0.0394). None of these effects on visits to the center were observed in females. *p<0.05 difference between designated groups.
